# Network Pharmacology-Based Strategy for Elucidating the Molecular Basis Forthe Pharmacologic Effects of Licorice (*Glycyrrhiza* spp.)

**DOI:** 10.3389/fphar.2021.590477

**Published:** 2021-04-28

**Authors:** Jia Chen, Lin-Fu Li, Xiao-Ru Hu, Feng Wei, Shuangcheng Ma

**Affiliations:** ^1^ School of Chinese Materia Medica, Beijing University of Chinese Medicine, Beijing, China; ^2^ Institute for Control of Chinese Traditional Medicine and Ethnic Medicine (ICCTMEM), National Institutes for Food and Drug Control (NIFDC), Beijing, China; ^3^ College of Pharmacy, Gannan Medical University, Ganzhou, China

**Keywords:** licorice, flavonoid, triterpenoid, pharmacologic effects, network pharmacology

## Abstract

Licorice (*Glycyrrhiza* spp.) is used widely in traditional Chinese medicine (TCM) due to its numerous pharmacologic effects. However, the mechanisms of action of the chemical constituents of licorice and their structure–function relationships are not fully understood. To address these points, we analyzed the chemical compounds in licorice listed in the TCM Systems Pharmacology database and TCM Integrated database. Target proteins of the compounds were predicted using Integrative Pharmacology-based Research Platform of TCM v2.0. Information on the pharmacologic effects of licorice was obtained from the 2020 *Chinese Pharmacopoeia*, and disease-related genes that have been linked to these effects were identified from the Encyclopedia of TCM database. Pathway analyses using the Kyoto Encyclopedia of Genes and Genomes database were carried out for target proteins, and pharmacologic networks were constructed based on drug target–disease-related gene and protein–protein interactions. A total of 451 compounds were analyzed, of which 211 were from the medicinal parts of the licorice plant. The 241 putative targets of 106 bioactive compounds in licorice comprised 52 flavonoids, 47 triterpenoids, and seven coumarins. Four distinct pharmacologic effects of licorice were defined: 61 major hubs were the putative targets of 23 compounds in heat-clearing and detoxifying effects; 68 were targets of six compounds in spleen-invigorating and qi-replenishing effects; 28 were targets of six compounds in phlegm-expulsion and cough-suppressant effects; 25 compounds were targets of six compounds in spasm-relieving and analgesic effects. The major bioactive compounds of licorice were identified by ultra-high-performance liquid chromatography–quadrupole time-of-flight–tandem mass spectrometry. The anti-inflammatory properties of liquiritin apioside, liquiritigenin, glycyrrhizic acid and isoliquiritin apioside were demonstrated by enzyme-linked immunosorbent assay (ELISA) and Western blot analysis. Liquiritin apioside, liquiritigenin, isoliquiritin, isoliquiritin apioside, kaempferol, and kumatakenin were the main active flavonoids, and 18α- and 18β-glycyrrhetinic acid were the main active triterpenoids of licorice. The former were associated with heat-clearing and detoxifying effects, whereas the latter were implicated in the other three pharmacologic effects. Thus, the compounds in licorice have distinct pharmacologic effects according to their chemical structure. These results provide a reference for investigating the potential of licorice in treatment of various diseases.

## Introduction

Licorice (referred toin Chinese as “GanCao”) is a food and dietary supplement that has been used widely in traditional Chinese medicine (TCM) for ∼4,000 years. According to the 2020 *Chinese Pharmacopoeia*, licorice refers to the dried root and rhizome of *Glycyrrhiza uralensis* Fisch. ex DC., *G. inflate* Bat., or *G. glabra* L.

As indicated in *Shennong’s Materia Medica Classic* (Shennong Bencao Jing), licorice is used predominantly to treat spleen dysfunction, stomach weakness, fatigue, lack of strength, palpitation, dyspnea, cough, profuse sputum, acute pain in the abdominal cavity, limb contracture, carbuncles, and sores, as well as to alleviate drug toxicity (Chinese Pharmacopoeia Commission, 2020). Because of its ability to “harmonize” the effects of different medicines, licorice is an ingredient in nine out of 10 herbal formulations.

The genus *Glycyrrhiza* comprises ∼30 species that are native to Eurasia and which have been cultivated in Europe (e.g., Spain, Italy, France), the Middle East (e.g., Syria, Iran, Turkey, Iraq) and Asia (e.g., China) (Chen J. et al., 2019). Eight species in the Leguminosae family are found in China: *G. uralensis* Fisch. ex DC.; *G. inflata* Bat.; *G. glabra* L.; *G. eurycarpa* P.C.Li; *G. aspera* Pall.; *G. yunnanensis* P.C.Li; *G. pallidiflora* Maxim.; *G. squamulosa* Franch. (Zeng et al., 1991; Karami et al., 2015). These species have various chemical constituents, such as flavonoids, triterpenoids, coumarins, and stilbenoids (Zhang and Ye, 2009; Cheng et al., 2012; Qiao et al., 2015).

There have been several reports on the pharmacologic effects and bioactive constituents of licorice, which are mainly flavonoids and triterpenoids (Wang et al., 2020). *In vivo* and *in vitro* experiments have shown that these classes of compound have anti-inflammatory, antimicrobial, antiviral, antioxidant, and antitumor effects. Recent studies have focused on the pathways regulating the pharmacologic effects of the metabolites of flavonoids and triterpenoids in licorice (Hosseinzadeh and Nassiri-Asl, 2015). However, given the diversity and complexity of the bioactive compounds in licorice, high-throughput and network-based approaches are needed to fully elucidate their pharmacologic properties and mechanisms of action.

Network pharmacology is an emerging discipline based on systems biology, analysis of biological networks, and identification of specific network nodes as targets in drug design (Hopkins, 2007; Li and Zhang, 2013). In general, treatment of diseases using TCM is based on integrative and holistic principles as well as the synergistic effects of multiple compounds and herbal formulations (Zhang et al., 2013). Network pharmacology adopts a similar holistic approach in aparadigm shift from “one target, one drug” to “network target, multi-compound” therapeutics (Li et al., 2014). Thus, network pharmacology is used widely to investigate the molecular mechanisms underlying the pharmacologic effects of TCM formulations (Li H. et al., 2014; Xiong et al., 2018; Piao et al., 2019); appropriate TCM prescriptions for the treatment of specific diseases (Li et al., 2007; Li X. et al., 2014; Shi et al., 2014; Ke et al., 2016; Fang et al., 2017a, 2017b; Hu and Sun, 2017; Dai et al., 2018; Shi et al., 2019); bioactive components of medicines (Lv et al., 2014; Zhang Y.-F. et al., 2019; Ma et al., 2019; Song et al., 2019; Guo et al., 2020).

Network pharmacology has been applied to studies on the mechanisms of action of TCM formulations containing licorice such as Sini, Shaoyao-Gancao, Guizhi-Shaoyao-Zhimu, Yinchensini, and Maxing-Ganshi decoctions, among others (Chen S. et al., 2014; Chen G. et al., 2018; Song et al., 2018; Zhang Q. et al., 2019; Zhu et al., 2019) as well as analyses of licorice constituents and their functions (Liu et al., 2013; Chen M. et al., 2019). The molecular mechanisms of licorice components in the context of diseases have also been studied (Li Y. et al., 2019). Those reports focused on compounds present in the overground and underground parts of the plant, which differ considerably (Wang, 2004; Zhou, 2015; Ran, 2019). The medicinal properties of licorice are associated mainly with the underground parts (roots and rhizomes).

Here, we classified the different types of bioactive compounds in the underground parts of the licorice plant. We also analyzed the mechanisms of action underlying the pharmacologic effects of licorice.

## Materials and Methods

### Chemicals, Reagents, and Materials

Ultra-high-performance liquid chromatography–mass spectrometry (UHPLC–MS)-grade acetonitrile and formic acid were supplied by Fisher Scientific (Fairlawn, NJ, United States). Ultrapure water (18.2MΩ) was prepared with a Milli-Q™ water-purification system (Millipore, Milford, MA, United States). All other reagents were of analytical grade and purchased from Sinopharm Chemical Reagents (Shanghai, China).

The reference compounds schaftoside (number 1; lot number, 111,912–201703; purity, 95.60%), calycosin 7-O-β-d-glucopyranoside (4; 111,920–201907; 96.80%), liquiritin (5; 111,610–201908; 95.00%), kaempferol (15; 110,861–202013; 93.20%), formononetin (17; 111,703–201504) and glycyrrhizic acid monoammonium salt (18; 110,731–202021; 96.20%) were supplied by National Institutes for Food and Drug Control (NIFDC; Beijing, China).

The reference compounds neoliquiritin (2; PRF20060,941; 97.88%), liquiritinapioside (3; PRF9050224; 99.95%), isoliquiritin apioside (6; PRF9101021; 97.04%), isoliquiritin (7; PRF20040,923; 98.23%), ononin (8; PRF20060,944; 99.58%), neoisoliquiritin (9; PRF20060,942; 99.25%), licochalcone B (10; PRF8031021; 99.10%), liquiritigenin (11; PRF20042,742; 99.50%), calycosin (12; PRF10072945; 99.70%), naringenin (13; PRF10030641; 99.67%), echinatin (14; PRF10122621; 99.81%), isoliquiritigenin (16; PRF20060,943; 99.87%), licoflavone A (19; PRF8050422; 99.94%), glycycoumarin (20; PRF20060,921; 99.77%), kumatakenin (21; PRF10120925; 99.28%), licochalcone A (25; PRF8041841; 98.65%), 18α-Glycyrrhetinic acid (29; PRF10101201; 99.02%) and 18β-Glycyrrhetinic acid (30; PRF9100841; 99.46%) were purchased from Chengdu BiopurifyPhytochemicals (Chengdu, China).

The reference compounds licoisoflavone A (23; PS010124; 98.46%), glycyrol (26; PS010089; 98.71%) and licoisoflavone B (28; PS200618–01; 98.02%) were obtained from Chengdu Push Biotech (Chengdu, China).

The reference compound licoflavonol (27; MUST-20041,311; 98.86%) was purchased from Chengdu Must Biotechnology (Chengdu, China). The reference compound licoricone (24; 200313G; 99.46%) was obtained from Nanjing Dasf Biotechnology (Nanjing, China). The reference compound licoflavone C (22; P29A9F68905; 99.30%) was purchased from Shanghai Yuanye Biotechnology (Shanghai, China).

Licorice materials were obtained from Elion Resources Group (Inner Mongolia, China) and Gansu JinYoukang Pharmaceutical Technology (Gansu, China). Licorice materials were authenticated as the dried roots of *Glycyrrhiza uralensis* Fisch. ex DC. by Professor Nanping Zhang (NIFDC, Beijing, China). The voucher numbers of licorice from Inner Mongolia wereN2-4-1 to N2-4-10, and from Gansu were G1-5-1 to G1-5-10. Voucher specimens were deposited in the Museum of Chinese Traditional Drugs within the NIFDC (Li X. et al., 2019).

### Preparation of Standard and Sample Solutions

Thirty reference compounds were prepared by completely dissolution in 70% methanol and their concentration (in mg/mL)was (compound 1) 0.266; 2) 0.187; 3) 0.178; 4) 0.416; 5) 0.258; 6) 0.186; 7) 0.269; 8) 0.299; 9) 0.166 (10) 0.155 11) 0.277 12) 0.189 13) 0.279 14) 0.222 15) 0.257 16) 0.214 17) 0.431 18) 0.227 19) 0.144 (20) 0.124 21) 0.144 22) 0.233 23) 0.263 24) 0.164 25) 0.221 26) 0.230 27) 0.186 28) 0.164 29) 0.172 (30) 0.337. All solutions were stored at 4°C before analyses.

All samples were pulverized and screened through the 50-mesh sieve. The dried powder (0.5g) was weighed accurately into a 100-ml conical flask with a stopper, and extracted by ultrasonication in 50ml of methanol (70%) for 0.5h. The mixture was centrifuged at 12,000rpm for 10min at room temperature. Finally, the supernatant was filtered through 0.22-μm membrane before injection into aUHPLC–MS/MS system.

### Ultra-High-Performance Liquid Chromatography–Quadrupole Time-of-Flight–Tandem Mass spectrometry(UHPLC–QTOF–MS/MS)

Chromatography was undertaken using an Acquity UHPLC HSS T3 C18 column (2.1mm i. d. × 100mm, 1.8μm) within an Acquity UHPLC system (Waters, Milford, MA, United States). The column temperature was maintained at 35°C. The mobile phase (at a flow rate of 0.4ml/min) consisted of solvent A (0.1%formic acid/water) and solvent B (acetonitrile). The conditions of gradient elution were optimized as: 5% B (0–1min), 5–18% B (1–3min), 18–30% B (3–13min), 30–45% B (13–18min), 45–50% B (18–21min), 50–75% B (21–29min), 75–95% B (29–31min), 95–5% B (31–31.5min) and held at 5% B for 3.5min to equilibrate the column. The injection volume was 2μL.

A Synapt^™^G2-S QTOF mass spectrometer (Waters MS Technologies, Manchester, UK) was combined with the UHPLC system via the electrospray-ionization source in positive-ion mode. The desolvation-gas rate was set as 600L/h at 500°C. The source temperature was set as 120°C. The capillary voltage was3 kV and the sample cone voltage was set at 30V. Centroided data were acquired from 50 to 1,000Da. Mass data were acquired using LockSpray™ to ensure the mass was recorded accurately. Leucine-enkephalin at a charge/mass ratio (*m/z*) 556.2771 was selected as the lock-mass in positive mode. The accurate mass and composition of precursor ions and fragment ions were calculated using MassLynx V4.1 (Waters), that incorporated with the instrument solution for the acquisition of accurate mass.

### Preparation of Bioactive Compounds

Four reference bioactive compounds, liquiritin apioside, liquiritigenin, glycyrrhizic acid and isoliquiritin apioside were prepared by completely dissolution in 0.1% DMSO/water and their concentration were 2mg/ml. The dried licorice extract powder (1.0g) was weighed accurately into a 100-ml conical flask with a stopper, and completely dissolved in 50ml of ultrapure water. All solutions were stored at −20°C before analyses.

### Cell Culture and Treatments

Murine macrophage RAW264.7 cells, a widely used *in vitro* model for studies of macrophage and inflammatory cascades, were obtained from the China National Collection of Authenticated Cell Cultures (Shanghai, China). Cells were maintained at 37°C under 5% CO_2_ in Dulbecco’s modified Eagle’s medium (DMEM) supplemented with 10% fetal bovine serum. RAW264.7 cells were treated with 1μg/ml lipopolysaccharide (LPS; Sigma-Aldrich; Merck KGaA, Darmstadt, Germany) for 24h at 37’ °C. The cells were rinsed with phosphate buffer saline (PBS) and stimulated with LPS (1ug/ml) to induce pyroptosis. Conditioned cells were collected for measurement of protein expression levels by ELISA and western blot analysis.

### ELISA Assay for Cytokines

Interleukin (IL)-1β and tumor necrosis factor (TNF)-α expression levels in the supernatant of treated cells were measured by ELISA assays (IL-1β: Mouse: ml063132-C; TNF-α: Mouse: ml002095-C; Enzyme-linked Biotechnology Co., Ltd., Shanghai, China) according to the manufacturer's instructions.

### Western Blot Analysis

The protein expression in RAW264.7 cells were detected by Western blot. Cells (1.6 × 10^5^ cells/well) were plated overnight and then treated with the indicated concentrations of bioactive compounds. After 1h, 1μg/ml LPS were added. Then, the supernatant and precipitation of cells were collected 24h later. Immunoblotting was performed using antibodies against the target proteins, including AKT1 (1:2,500; ab89402; Abcam, Cambridge, MA, United States), p-AKT1 (1:5,000; ab81283; Abcam, Cambridge, MA, United States), PI3K (1:1,000; 4,249; Cell Signaling Technology, Inc., Danvers, MA, United States), p-PI3K (1:1,000; bs-3332R; Bioss Antibodies, Biotechnology, Inc., Beijing, China), NFκB-p65 (1:1,000; sc-8008; Santa Cruz Biotechnology, Inc.,Dallas, TX, United States), p-NFκB-p65 (1:1,000; YP0191; Immuno Way, Biotechnology, Inc., Plano, TX, United States). The blots were developed with an enhanced chemiluminescence kit (ECL, Amersham Biosciences, Buckinghamshire, United Kingdom) and measured by using a luminescent image analyzer (LAS-3000, Fuji Photo Film Co. Ltd., Japan).

### Statistical Analyses

All experimental values were presented as the mean ± standard error of the mean. Statistical comparison between two groups was performed by Student’s *t*-test, and one-way analysis of variance followed by Bonferroni *post hoc* analyses was performed among multiple groups for parametric data. *P* < 0.05 was considered to indicate a statistically signifcant difference.

### Chemical Compounds in Licorice

Candidate chemical compounds in licorice were searched in two phyto chemical databases: Traditional Chinese Medicine Systems Pharmacology (TCMSP; http://tcmspw.com/tcmsp.php) (Ru et al., 2014) and Traditional Chinese Medicines Integrated database (TCMID; www.megabionet.org/tcmid/). Components of the underground parts of the licorice plant were screened from candidate compounds by reviewing the literature. National Center for Biotechnology Information (NCBI) PubMed (https://pubmed.ncbi.nlm.nih.gov/) and China National Knowledge Infrastructure (CNKI; www.cnki.net/) databases were used to find the underground parts of licorice. In the PubMed database, “licorice chemical composition” were used as keywords to search for studies from 2000 to 2020. In the CNKI database, we searched for doctor altheses from 2000 to 2020 under the keyword of “licorice”. Two-dimensional (2D) chemical structures were obtained from NCBI PubChem (https://pubchem.ncbi.nlm.nih.gov/) and SciFinder Scholar (https://scifinder.cas.org/scifinder/) databases. If a structure could not be retrieved from these databases, the original research article describing the identification or purification of the compound was searched for. ChemBioDraw Ultra v12.0 (PerkinElmer, Waltham, MA, United States) was used to draw structures, which were saved in sdf or mol2 formats (Xiong et al., 2019).

### Putative Targets of Bioactive Compounds in Licorice

The sdf or mol2 files were uploaded to Integrative Pharmacology-based Research Platform of Traditional Chinese Medicine (TCMIP) v2.0 (www.tcmip.cn/). The putative targets of the compounds in licorice were predicted using the drug target-prediction tool of TCMIP. We selected only pairs of compound–putative targets in which the structural similarity score of the compound to known drugs was >0.80 (moderate–high similarity). Detailed information on putative targets are shown in Supplementary Table S1.

### Disease-Related Genes Associated with Thepharmacologic Effects of Licorice

Disease-related genes associated with the pharmacologic effects of licorice were identified from the Encyclopedia of Traditional Chinese Medicine database (www.nrc.ac.cn:9090/ETCM) (Xu et al., 2019). The genes related to “immune inflammation” imbalance and some psychiatric symptoms were obtained as disease/symptom gene sets corresponding to heat-clearing and detoxifying effects. The genes related to anemia, low resistance, mental disorders, organ dysplasia, reproductive capacity and other symptoms were obtained as disease/symptom gene sets corresponding to spleen-invigorating and qi-replenishing effects. The genes related to the throat, trachea, bronchus, inflammation, lung disease, asthma and other symptoms were obtained as disease/symptom gene sets corresponding to phlegm-expulsion and cough-suppressant effects. The genes related to the chest, abdomen, limb-spasm pain and neurological diseases were obtained as the disease/symptom gene sets corresponding to spasm-relieving and analgesic effects.

### Network of Interactions Between Drug Targets and Disease-Related Genes

Networks of interactions between drug targets and disease-related genes were constructed based on the relationships between the putative targets of compounds in licorice and disease-related genes associated with the pharmacologic effects of licorice. Construction and analyses of such networks were carried out with TCMIP v2.0 (www.tcmip.cn). Visualization of such networks was done using Navigator v2.2.1 (Krembil Research Institute, Toronto, ON, Canada). Hubs with a degree greater than two fold the median value of all node degrees were screened, and a network was constructed based on direct interactions between hubs. Three topologic properties of the hub network (degree, betweenness, and closeness) were calculated to identify targets of topologic importance. Major hubs were identified as those with network topology values that were higher than the corresponding median values.

### Analyses of Pathway Enrichment

Pathway-enrichment analyses were done using database Visualization and Integrated Discovery v6.7 (http://david.abcc.ncifcrf.gov/home.jsp) based on pathway data obtained from the Kyoto Encyclopedia of Genes and Genomes (KEGG)database (www.genome.jp/kegg; updated on 18 November 2016) (Kanehisa and Goto, 2000; Dennis et al., 2003). Only functional annotations with enrichment *P*-values corrected with Bonferroni and Benjamini algorithms (*p* < 0.05) were selected for further analyses.

## Results and Discussion

### Analyses of Licorice Composition and Compound Screening

A total of 451 compounds were screened from TCMSP and TCMID (Supplementary Table S2). Of these, 211 were identified by literature review as compounds present in the medicinal portion of thelicorice plant:134 flavonoids, 49 triterpenoids, 18 coumarins, and 10 stilbenoids (Supplementary Table S3). Most network-pharmacology studies on licorice have used a single database to identify itschemical constituents. We compared compounds in TCMSP and TCMID, which are used widely in TCM research and provide the names and 2D structures of compounds. We found that some were commonto both databases whereas others were unique. Some compounds were duplicated in the same database. We obtained 604 compounds from the initial search (280 from TCMSP and 324 from TCMID). We found that 26 compounds were duplicated in TCMID and, therefore, there were actually 298 chemical compounds; 127 were common to both TCMSP and TCMID. Compared with earlier network-pharmacology studies of licorice, our screen of the chemical constituents of licorice was more comprehensive and allowed more accurate prediction of molecular mechanisms. The compounds were grouped according to their2D structure for further analyses of the relationship between the type of compound and pharmacologic effects of licorice.

### Putative Targets of Compounds in Licorice

Based on their structural similarity to known chemical compounds, 241 putative targets were identified for 106 chemical compounds in licorice: 52 targeted by flavonoids, 47 by triterpenoids, and seven by coumarins. Detailed information on the bioavailability of these chemical compounds is provided in Supplementary Table S4 (Ru et al., 2014; Daina et al., 2017; Xu et al., 2019). Enrichment analyses based on the biological pathways in the KEGG database revealed that the putative targets were mainly involved in neuromodulation [neuroactive ligand–receptor interactions (*P* = 3.90 × 10^–10^), long-term potentiation (*P* = 0.001), and gap junctions (*P* = 0.03)], energy production and metabolic pathways (nitrogen metabolism (*P* = 7.84 × 10^–10^), linoleic-acid metabolism (*P* = 1.30 × 10^–7^) and oxidative phosphorylation (*P* = 9.47 × 10^–4^) and inflammation/immune-system regulation (Fc epsilon RI (*P* = 0.01), toll-like receptor (TLR; *P* = 0.01) and nucleotide-binding and oligomerization domain-like receptor (NLR; *P* = 0.02) signaling pathways.

### Pharmacologic Mechanisms of Compoundsin Licorice

#### Pharmacologic Mechanisms of Heat-Clearing and Detoxifying Effects

Of the 241 putative targets, 37 were disease-related genes involved in the heat-clearing and detoxifying effects of licorice. The network of interactions of drug target–disease-related genes contained 985 nodes and 3,974 interactions. A total of 299 major hubs were identified based on the values of three topologic features of the network (node degree, betweenness, and closeness). Of these, 61 hubs were the putative targets of 23 chemical compounds in licorice: echinatin, glabrolide, glycycoumarin, glycyrol, glycyrrhizic acid, isoliquiritin, kumatakenin, licoarylcoumarin, licochalcone B, licoricesaponin A3, licoricesaponin B2, licoricesaponin C2, licoricesaponin D3, licoricesaponin E2, licoricesaponin F3, licoricesaponin G2, licoricesaponin J2, licoricesaponin K2, liquiritigenin, liquiritin, methyl glycyrrhetate, neoisoliquiritin, and ononin.

To investigate the mechanisms underlying the heat-clearing and detoxifying effects of licorice, a network was constructed based on direct interactions between major hubs that was divided into four functional modules.

The first nodule was regulation of the balance between inflammation and the immune system. This comprised the genes involved in nuclear factor-kappa B(NF-κB) (Zhang et al., 2015), tumor necrosis factor (TNF) (Graßmann et al., 2017), TLR (Belforte et al., 2013), and NLR signaling pathways (Luan et al., 2018), as well as inflammatory regulation of transient receptor potential (TRP) channels (Sahoo et al., 2019).

The second nodule was modulation of the nervous system. This comprised the genes involved in retrograde endocannabinoid signaling (Musella et al., 2017), neuroactive ligand–receptor interactions (Schüle et al., 2014), γ-aminobutyric acid [GABA]ergic (Streeter et al., 2010) as well as serotonergic synapses and neurotrophin signaling pathways (Kashyap et al., 2018).

The third nodule was regulation of energy production and metabolism. This comprised the genes involved in adipocytokine (Kita et al., 2019) and thyroid-hormone signaling pathways (Mullur et al., 2014).

**FIGURE 1:**
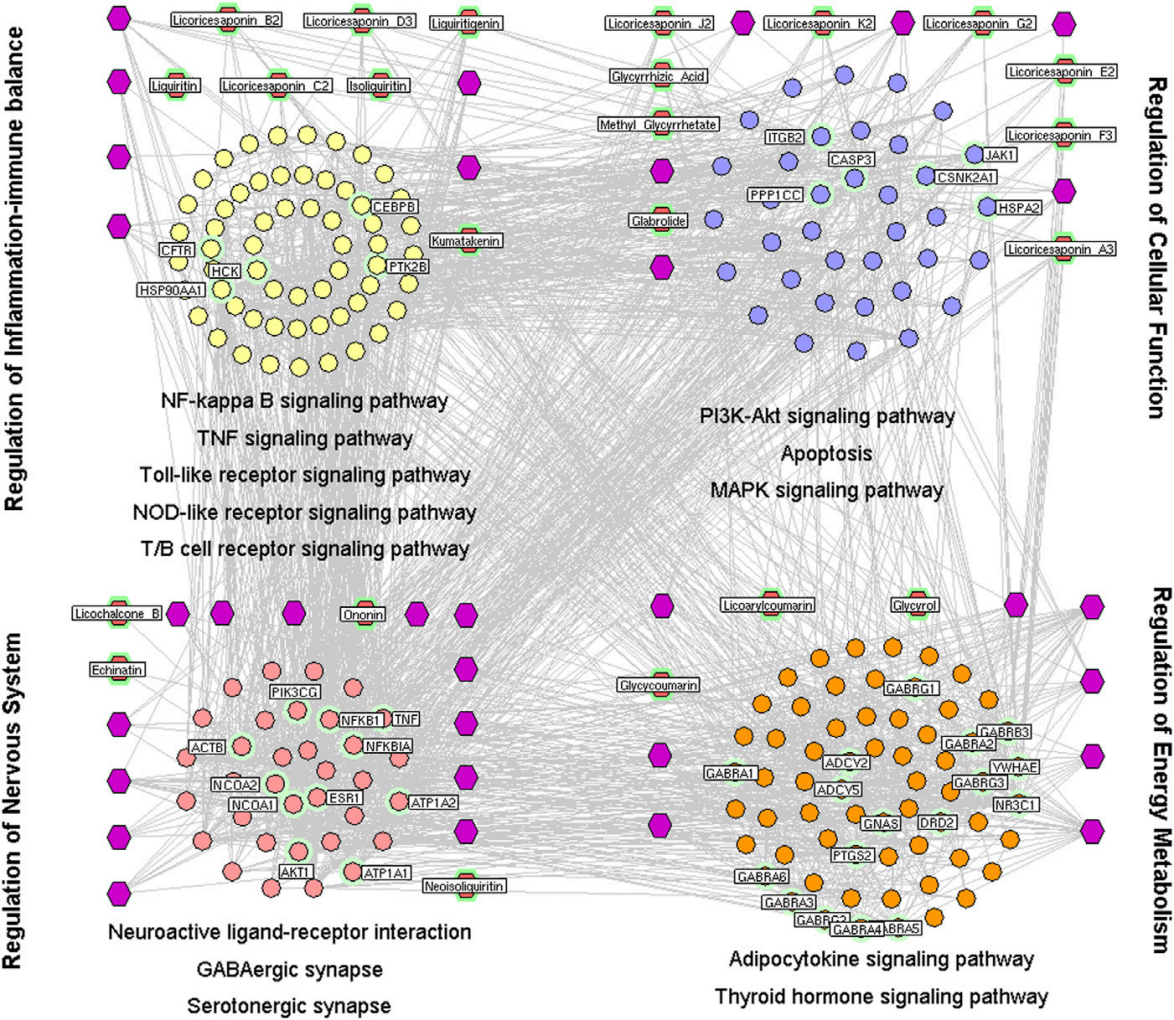
Interaction network of chemical compounds containing licorice and the corresponding major targets associated with its heat-clearing and detoxifying effects. To investigate the mechanisms underlying the heat-clearing and detoxifying effects of licorice, a network was constructed based on direct interactions between major hubs that was divided into four functional modules. Purple hexagons represent chemical components, red hexagons denote representative the chemical components of licorice, yellow circles denote the core targets related to regulation of balance of inflammation and the immune system, and blue circles represent the core targets related to cellular functions. The pink circle represents the core target related to modulation of the nervous system, the orange circle represents the core target related to regulation of energy production and metabolism.

The fourth nodule was cellular functions. This comprised genes involved in phosphoinositide 3-kinase/protein kinase B ([PI3K/AKT) (Hong et al., 2016) and mitogen-activated protein kinase (MAPK) signaling pathways (Park, 2018) and apoptosis (Kawamoto et al., 2016) (Figure 1).

#### Pharmacologic Mechanisms of Spleen-Invigorating and Qi-Replenishing Effects

Of the 241 putative targets, 78 were disease-related genes associated with the spleen-invigorating and qi-replenishing effects of licorice. The network of interactions of drug target–disease-related genes contained 1,729 nodes and 9,181 interactions. Based on the node degree, betweenness, and closeness values, 534 major hubs were identified, of which 68 were the putative targets of six compounds (18α-and 18β-glycyrrhetinic acid, isoliquiritin apioside, kumatakenin, licoarylcoumarin, and liquiritin apioside).

We wished to investigate the mechanisms underlying the spleen-invigorating and qi-replenishing effects of licorice. Hence, a major hub network was constructed based on the direct interactions between major hubs that were divided into four functional modules.

The first functional node was regulation of balance of inflammation and the immune system. This comprised genes involved in PI3K/AKT, MAPK, TNF, Fc epsilon RI (Ben Mkaddem et al., 2019), and NF-κB signaling pathways, and inflammatory regulation of TRP channels.

The second functional node was regulation of energy production and metabolism. This was composed of genes involved in thyroid hormone, cyclic (c)AMP (Alqurashi et al., 2016), insulin (Guo and Guo, 2017), adipocytokine, and 5ʹ AMP-activated protein kinase (Ke et al., 2018) signaling pathways.

The third functional module was modulation of the nervous system. This comprised genes involved in the neurotrophin signaling pathway, gap junctions (Dong et al., 2018), long-term potentiation/depression (Bliss and Cooke, 2011), and neuroactive ligand–receptor interactions.

**FIGURE 2:**
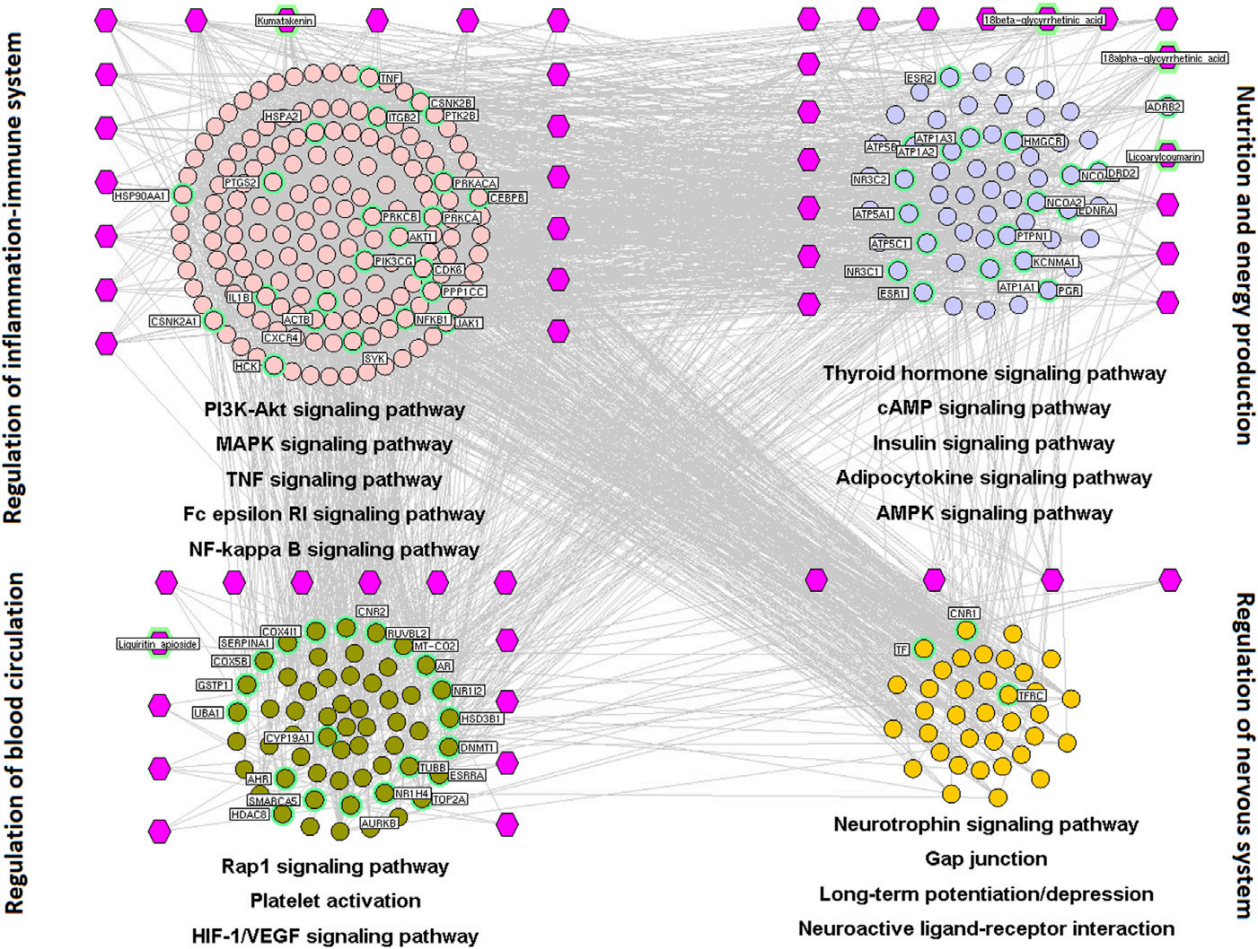
Interaction network of chemical compounds containing licorice and the corresponding major targets associated with spleen-invigorating and qi-replenishing effects. We wished to investigate the mechanisms underlying the spleen-invigorating and qi-replenishing effects of licorice. Hence, a major hub network was constructed based on the direct interactions between major hubs that were divided into four functional modules. Purple hexagons represent chemical components, pink circles denote core targets related to regulation of balance of inflammation and the immune system, blue circles represent core targets related to nutrition and energy production, green circles denote core targets related to regulation of blood circulation, and the orange circle represents the core target related to modulation of the nervous system.

The fourth functional module was regulation of angiogenesis and circulation. This comprised genes involved in Ras-associated protein (Rap)1 (Rho et al., 2017), hypoxia-inducible factor (HIF)-1 (Semenza, 2014), and vascular endothelial growth factor (VEGF) signaling pathways (Gianni-Barrera et al., 2014) as well as contraction of vascular smooth muscle and platelet activation (Starlinger et al., 2011) (Figure 2).

#### Pharmacologic Mechanisms of Phlegm Expulsion and Cough-Suppressant Effects

Of the 241 putative targets, nine were disease-related genes involved in the phlegm-expulsion and cough-suppressant effects of licorice. The network of interactions between drug targets and disease-related genes contained 301 nodes and 851 interactions. Ninety-seven major hubs were identified based on the values of the node degree, betweenness, and closeness in the network. Of these, 28 were the putative targets of six compounds in licorice (18α- and 18β-glycyrrhetinic acid, isoliquiritin apioside, kumatakenin, licoarylcoumarin, and liquiritin apioside).

We wished to investigate the mechanistic basis of the phlegm-expulsion and cough-suppressant effects of licorice. A major hub network was constructed based on the direct interactions between major hubs that was divided into three functional modules.

The first functional nodule was regulation of the balance between inflammation and the immune system. This comprised the genes involved in: TNF, NF-κB, T-/B-cell receptors (Lee and Korner, 2019), NLR, and TLR signaling pathways; leukocyte transendothelial migration (Nourshargh and Alon, 2014); *tuberculosis* (Khader et al., 2019); pertussis (Murphy et al., 2020).

The second functional nodule was regulation of energy production and metabolism. This was composed of the genes involved in thyroid hormone, cAMP, adipocytokine, and glucagon signaling pathways (Kleinert et al., 2019); regulation of lipolysis in adipocytes (Shin et al., 2017) as well asthyroid-hormone synthesis.

**FIGURE 3:**
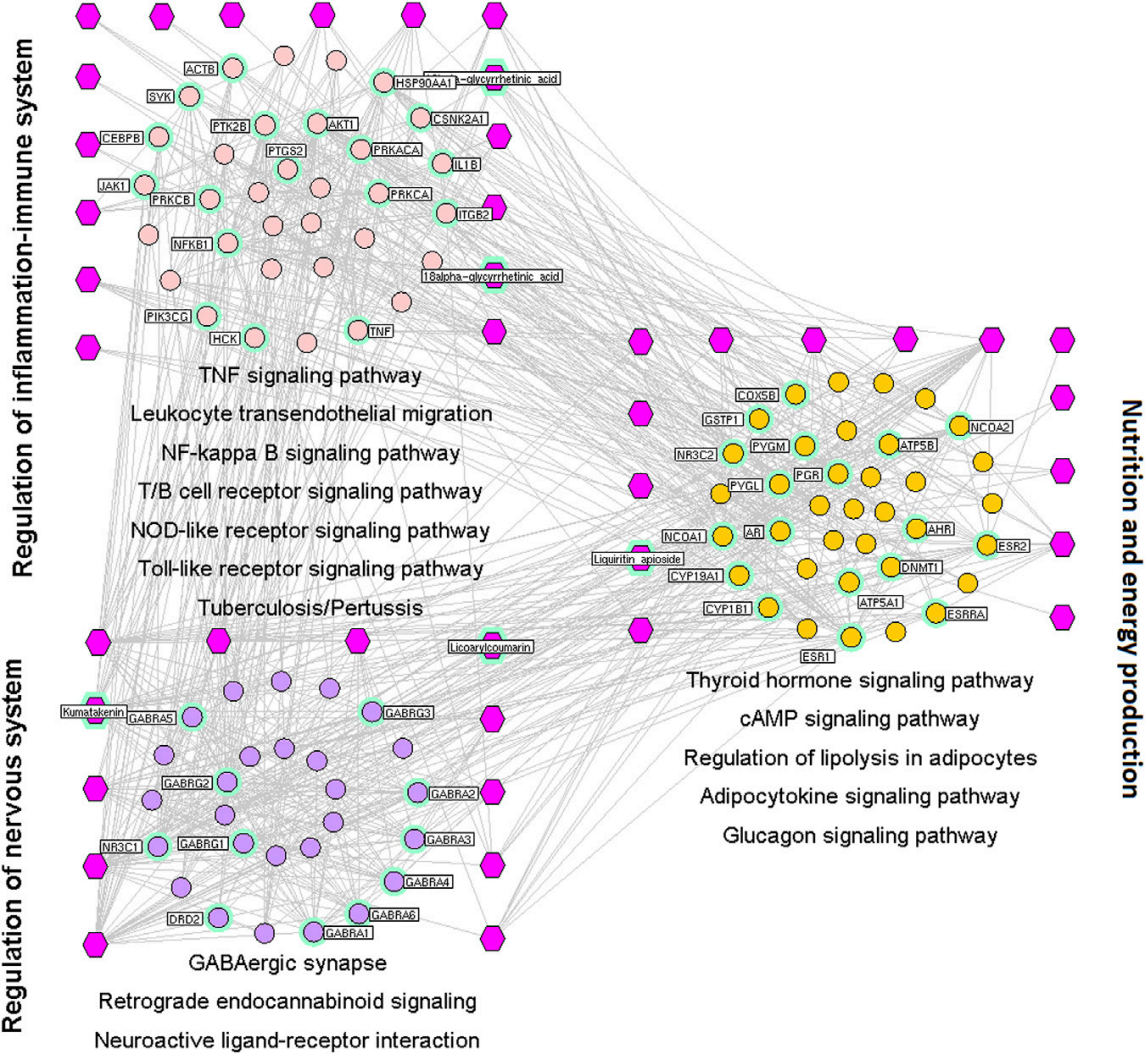
Interaction network of chemical compounds containing licorice and the corresponding major targets associated with phlegm-expulsion and cough-suppressant effects. We wished to investigate the mechanistic basis of the phlegm-expulsion and cough-suppressant effects of licorice. A major hub network was constructed based on the direct interactions between major hubs that was divided into three functional modules. Purple hexagons represent chemical components, pink circles denote core targets related to regulation of balance of inflammation and the immune system, light purple circles represent core targets related to modulation of the nervous system, and orange circles denote core targets related to nutrition and energy production.

The third functional nodulewas modulation of the nervous system. This comprised genes involved in GABAergic synapses (Babaev et al., 2018); retrograde endocannabinoid (Lu and Mackie, 2017) and chemokine (Zigmond and Echevarria, 2019) signaling pathways, as well as neuroactive ligand–receptor interactions (MacKenzie EM et al., 2007) (Figure 3).

#### Pharmacologic Mechanisms of Spasm-Relieving and Analgesic Effects

Of the 241 putative targets, 29 were disease-related genes linked to the spasm-relieving and analgesic effects of licorice. The network of interactions of the drug target and disease-related genes contained 689 nodes and 2,682 interactions. Based on the network topology values of node degree, betweenness, and closeness, 200 major hubs were selected, of which 25 were the putative targets of six compounds in licorice (18α- and 18β-glycyrrhetinic acid, isoliquiritin apioside, kumatakenin, licoarylcoumarin, and liquiritin apioside).

We wished to investigate the mechanisms associated with the spasm-relieving and analgesic effects of licorice. Hence, a network was constructed based on the direct interactions between major hubs that were divided into three functional modules.

The first functional module was modulation of neuroinflammation and neuropathologic pain. This comprised thegenes involved in MAPK, calcium (Navakkode et al., 2018), TNF, and TLR signaling pathways, as well as neuroactive ligand–receptor interactions (Salvalaio et al., 2017).

The second functional module was regulation of energy production and metabolism. This was composed of the genes involved in cAMP and adipocytokine signaling pathways, regulation of lipolysis in adipocytes, and oxidative phosphorylation (Bald et al., 2017).

**FIGURE 4:**
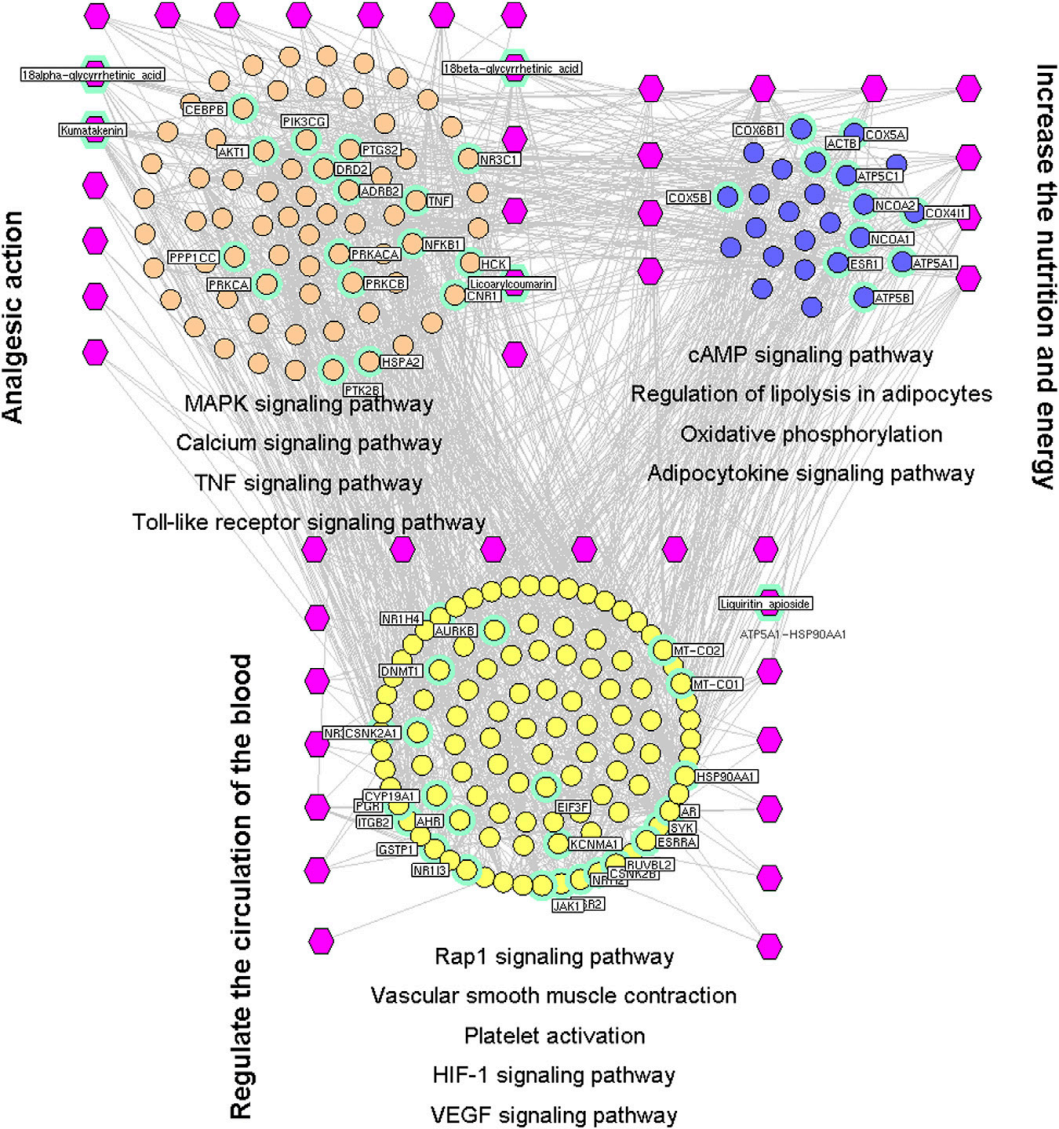
Interaction network of chemical compounds containing licorice and the corresponding major targets associated with spasm-relieving and analgesic effects. We wished to investigate the mechanisms associated with the spasm-relieving and analgesic effects of licorice. Hence, a network was constructed based on the direct interactions between major hubs that were divided into three functional modules. The purple hexagon represents the chemical composition, the pink circle denoted the core target related to analgesic action, the blue circle represents the core target related to nutrition and energy production, and the yellow circle denoted the core target related to regulation of blood circulation.

The third functional module was regulation of angiogenesis and circulation. This comprised the genes involved in Rap1, HIF-1 and VEGF signaling pathways as well as contraction of vascular smooth muscle and platelet activation (Figure 4).

### Analyses of the Main Bioactive Ingredients of Licorice

**FIGURE 5:**
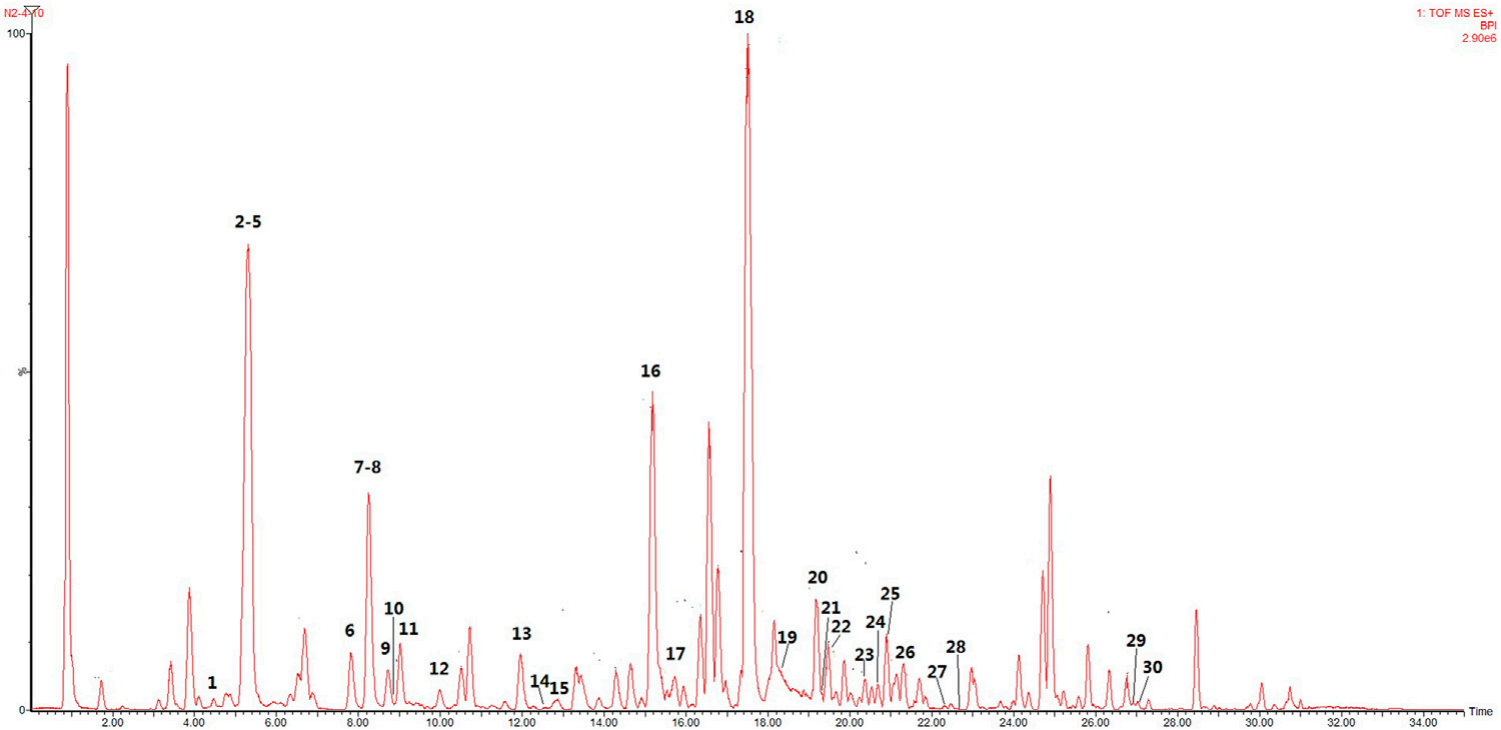
Representative base peak intensity (BPI) chromatograms of licorice derived from UHPLC–QTOF–MS/MS.

**FIGURE 6:**
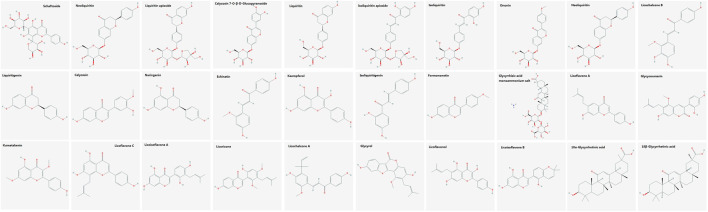
Two-dimensional chemical structures of the bioactive compounds in licorice.

The main bioactive components of licorice could be detected by UHPLC–TOF–MS/MS (Figure 5). Thirty bioactive compounds were identified by comparing the retention time and quasi-molecular ions with reference standards, respectively. The structures of these compounds are shown in Figure 6. Information such as retention time (min), CAS number, molecular formula, *m/z*, and MS^2^ fragments is offered in Supplementary Table S5.

Liquiritin apioside and liquiritigenin were the main flavonoids, and 18α- and 18β-glycyrrhetinic acid were the main triterpenoids, in licorice. These compounds were found to be implicated in the various pharmacologic effects of licorice. Liquiritin apioside, which was also present at a high level in licorice, can be used as a marker for the quality assessment of *Glycyrrhiza* species (Jiang et al., 2016). The phlegm-expulsion and cough-suppressant effects of liquiritin apioside have been reported (Kuang et al., 2018; Wei et al., 2020). Also, liquiritin apioside has been linked to TNF, NF-κB, NLR, TLR, and adipocytokine signaling pathways, which have important roles in anti-inflammatory immune activity, energy production, and metabolism (Guan et al., 2012).

Liquiritigenin has potent pharmacologic activity, including inhibitory effects on fibrogenesis and inflammation in the liver (Huang et al., 2019; Lee et al., 2019). We predicted the following proteins to be the putative targets of liquiritigenin in the heat-clearing and detoxifying effects of licorice: AKT1, cyclin-dependent kinase 6, heat-shock protein (HSP)90AA1, HSPA2, Janus kinase, phosphatidylinositol-4,5-bisphosphate 3-kinase catalytic subunit γ, casein kinase 2α1, CCAAT enhancer-binding protein β, β-actin, estrogen receptor 1, nuclear receptor coactivator (NCOA)1, and NCOA2. Our analyses suggested that liquiritigenin exerts heat-clearing and detoxifying effects *via* regulation of PI3K/AKT (Shi et al., 2015; Tao et al., 2016; Meng and Lin, 2019), MAPK (Tu et al., 2019), NF-κB (Zhu et al., 2018), TNF (Yu et al., 2015), and neurotrophin (Liu et al., 2009)signaling pathways and apoptosis (Bae et al., 2018).

18α-and 18β-glycyrrhetinic acid are representative triterpenoid saponins present in high concentrations in licorice (Wang and Yang, 2007). They have anti-inflammatory, antiviral, hepatoprotective, and anti-tumor effects. We predicted the following proteins to be the putative targets of 18α-and 18β-glycyrrhetinic acid in the spleen-invigorating and qi-replenishing effects of licorice: NF-κB subunit 1, estrogen receptor1, NCOA1, NCOA2, 3-mydroxy-3-methylglutaryl-coA reductase, integrin subunit β2, and nuclear receptor subfamily 3 group C member 1. These proteins constituted two functional modules. The first functional module was regulation of balance of inflammation and the immune system, with genes involved in PI3K/AKT (Kao et al., 2010; Wang et al., 2011), MAPK (Chen X. et al., 2018; Zhang Y. et al., 2019), TNF (Zhou and Wink, 2019) and NF-κB (Cao et al., 2017) signaling pathways. The second functional module was regulation of energy production and metabolism (Chang et al., 2010, Chen et al., 2014a), with genes involved in thyroid hormone, cAMP, and adipocytokine signaling pathways (Shamsa et al., 1991; Rastegari et al., 2019).

Liquiritin and glycyrrhizic acid are the main representative chemical components of licorice (Chinese Pharmacopoeia Commission, 2020). We found that liquiritin and glycyrrhizic acid had putative targets, but the number of their putative targets were not as high as those of liquiritin apioside, 18β-glycyrrhetinic acid or other chemical components. The pharmacologic effects of the chemical components of licorice were related not only to the strength of biological activity but also to their concentration. Chemical components with strong biological activity but very low concentration contributed little to the pharmacologic effect. However, the chemical components with moderate activity but very high concentration contributed considerably to the pharmacologic effect. Biological activity and concentration are the most important factors for selecting quality control (QC) markers. We believe that liquiritin and glycyrrhizic acid should be used as QC markers for licorice.

### Main Bioactive Ingredients of Licorice Reduce the Inflammatory Responses

Based on the result of network pharmacology, liquiritin apioside, liquiritigenin and isoliquiritin apioside were found as the main bioactive components of licorice. Liquiritin apioside and isoliquiritin apioside comprised the genes involved in NF-κB, TNF and IL-1β, while liquiritigenin comprised the genes involved in PI3K/AKT. Glycyrrhizic acid is the main representative chemical components of licorice which comprised the genes involved in NF-κB and TNF. All of these genes have important roles in anti-inflammatory immune activity.

**FIGURE 7:**
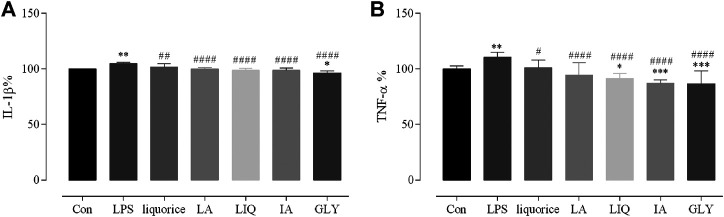
Reduce effects of the main bioactive compounds of licorice on the LPS-induced production of cytokines and chemokines. The supernatants were collected for measuring the levels of IL-1β (A), TNF-α (B) by ELISA. Data are presented as the mean ± standard error of the mean (*n* = 3). *p < 0.05, **p < 0.01, ***p < 0.001, ****p < 0.001 vs. Con group; #*p* < 0.05, ##*p* < 0.01, ###*p* < 0.001, ####*p* < 0.0001 vs. LPS group. Liquiritin apioside, LA; Liquiritigenin, LIQ; Isoliquiritin apioside, IA; Glycyrrhizin, GLY; IL, interleukin; LPS, lipopolysaccharide; TNF, tumor necrosis factor.

To determine whether the main bioactive components of licorice can reduce the inflammatory response, the levels of TNF-α and IL-1β were measured in RAW 264.7 by ELISA analysis. Liquiritin apioside, liquiritigenin, glycyrrhizic acid and isoliquiritin apioside were chosen as representative bioactive components. In the vitro experiment, the levels of TNF-α and IL-1β in RAW 264.7 were significantly increased after stimulation with LPS compared with those in the blank control (*P* < 0.01). The LPS group showed higher levels of TNF-α and IL-1β than those of the four bioactive components-treated and licorice-treated LPS group (*P* < 0.05), indicating that the excessive secretion of TNF-α and IL-1β induced by LPS could be reduced by liquiritin apioside, liquiritigenin, glycyrrhizic acid, isoliquiritin apioside and licorice (Figure 7). According to these results, we found that the inflammatory responses could be reduced by the main bioactive components of licorice treatment.

**FIGURE 8:**
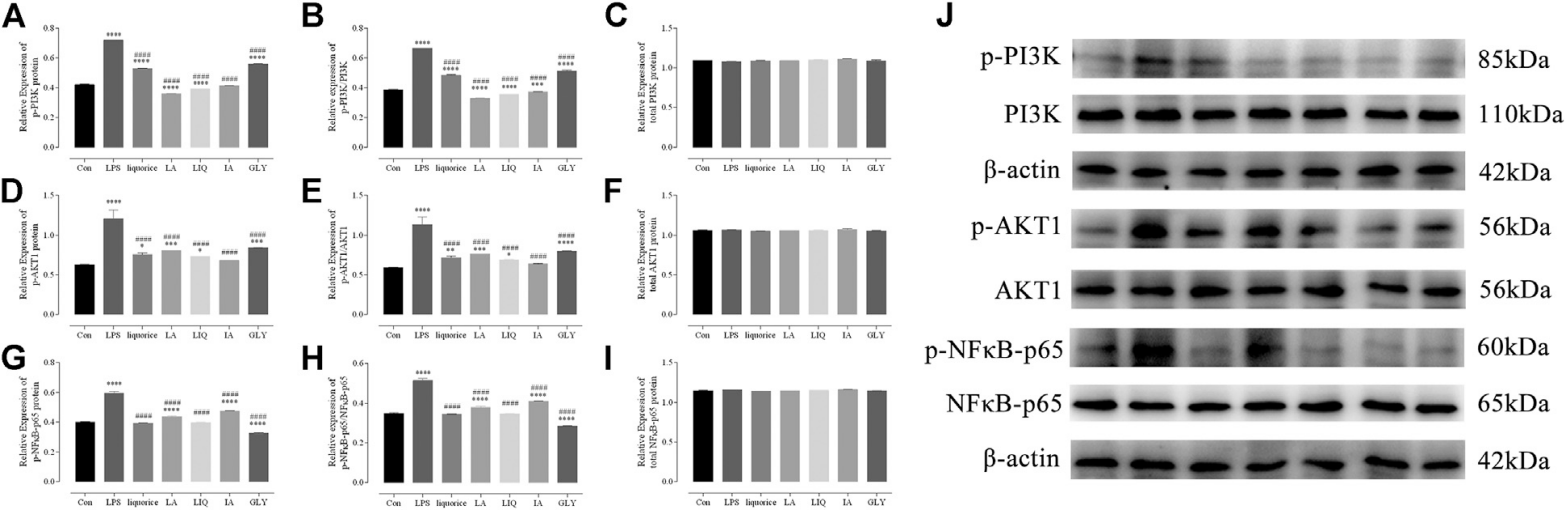
Reduce effects of the main bioactive compounds of licorice on the PI3K/AKT/NFκB signaling pathway. The levels of PI3K, AKT and NF-κB proteins were detected by Western blotting(J). The relative expression of p-PI3K(A), p-AKT1(D), p-NFκB-p65(G) protein and ratio of p-PI3K/PI3K(B), p-AKT1/AKT1(E), p-NFκB-p65/NFκB-p65(H) and total PI3K(C), AKT1(F) and NFκB-p65/NFκB-p65(I) protein were quantified. Data are presented as the mean ± standard error of the mean (*n* = 3). *p < 0.05, **p < 0.01, ***p < 0.001, ****p < 0.001 vs. Con group; #*p* < 0.05, ##*p* < 0.01, ###*p* < 0.001, ####*p* < 0.0001 vs. LPS group. Liquiritin apioside, LA; Liquiritigenin, LIQ; Isoliquiritin apioside, IA; Glycyrrhizin, GLY; LPS, lipopolysaccharide.

In order to investigate the mechanisms underlying the anti-inflammatory activities of bioactive components of licorice, Western blotting analysis was used to verify the regulatory effects of the main bioactive components. The results of Western blotting revealed that bioactive components are able to markedly suppressed the PI3K/AKT/NFκB signaling pathway, mediated by p-PI3K (Figure 8A), p-AKT1 (Figure 8D), p-NFκB-p65 (Figure 8G), p-PI3K/PI3K (Figure 8B), p-PI3K/PI3K (Figure 8B), p-AKT1/AKT1 (Figure 8E) and p-NFκB-p65/NFκB-p65 (Figure 8H) in LPS-activated macrophages.

The PI3K/AKT/NFκB signaling pathway plays an important role in the regulation of signal transduction and biological processes such as cell proliferation, apoptosis, metabolism and angiogenesis. The regulatory echanisms and biological functions of the PI3K/AKT/NFκB signaling pathway are important in many human diseases, including ischemic brain injury, neurodegenerative diseases, and tumors and play an important role in erythropoiesis and glycolysis (Xie et al., 2019; Xu et al., 2020). Nuclear factor-kappaB (NF-κB) proteins constitute a family of transcription factors that are stimulated by pro-inflammatory cytokines, chemokines, stress-related factors and extracellular matrix (ECM) degradation products, which has long been considered a prototypical proinflammatory signaling pathway (Toby, 2009; Stella and Athanasios, 2013). These results indicat that the main bioactive components of licorice inhibit the expression of TNF-α and IL-1β in the downstream through the PI3K/AKT/NFκB signaling pathway. Our results further demonstrate that the main bioactive components of licorice have anti-inflammatory properties.

### Functions of Different Types of Compound in the Pharmacologic Effects of Licorice

We analyzed the major targets of the chemical compounds associated with the pharmacologic effects of licorice. We showed that the activities of the compounds varied according to their chemical structure, with flavonoids and triterpenoids having the most important role, data that are consistent with results from the work of Wang and colleagues (Wang et al., 2020).

**FIGURE 9:**
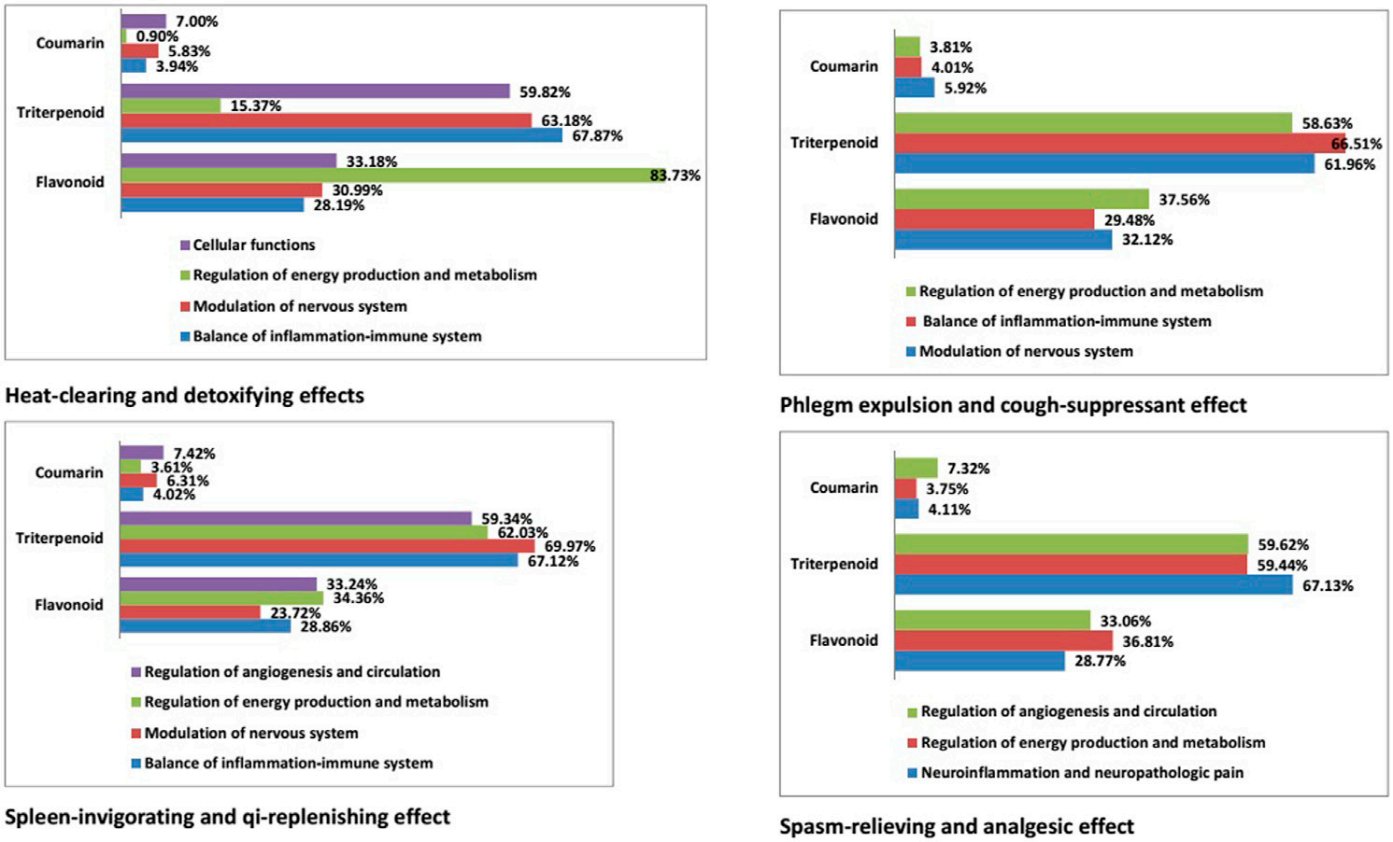
Proportion of different chemical structure types of licorice in various pharmacologic effects.

We constructed a network for the heat-clearing and detoxifying effects of licorice comprising four functional modules: cellular functions; modulation of the nervous system; balance between inflammation and the immune system; regulation of energy production and metabolism. Triterpenoids were the predominant type of compound in the first three modules, whereas flavonoids had a leading role in regulation of energy production and metabolism (accounting for 83.73% of compounds within this module). Triterpenoids participated to varying degrees in the different functional modules. In the spleen-invigorating and qi-replenishing effects of licorice, they were important formodulation of the nervous system (69.97%). In the phlegm-expulsion and coughing-suppressant effects, they were responsible for regulating the balance between inflammation and the immune system (66.51%). In the spasm-relieving and analgesic effects, they played a major part in neuroinflammation and neuropathologic pain (67.13%) (Figure 9).

## Conclusion

The pharmacologic effects of licorice arise from the combined action of various types of compounds. Triterpenoids and flavonoids make the greatest contribution, but coumarins are also important. Each pharmacologic effect of licorice comprised multiple functional modules. This finding is consistent with the general premise of TCM that therapeutic mechanisms involve many compounds and targets. We suggest that, for invigorating the spleen and replenishing qi, expelling phlegm and suppressing cough, or relieving spasm and pain, licorice with a higher triterpenoid content may be used. Licorice with higher levels of flavonoids may be more appropriate for heat-clearing and detoxification. Our results provide a reference for the QC of licorice and for investigating its therapeutic potential in the treatment of specific symptoms or diseases.

## Data Availability

The original contributions presented in the study are included in the article/Supplementary Material, further inquiries can be directed to the corresponding authors.
